# Pharmacological Manipulation of Wnt/β-Catenin Signaling Pathway in Human Neural Precursor Cells Alters Their Differentiation Potential and Neuronal Yield

**DOI:** 10.3389/fnmol.2021.680018

**Published:** 2021-08-04

**Authors:** Michael Telias, Dalit Ben-Yosef

**Affiliations:** Wolfe PGD-SC Lab, Racine IVF Unit, Department of Cell and Developmental Biology, Lis Maternity Hospital, Tel-Aviv Sourasky Medical Center, Sackler Medical School, Tel-Aviv University, Tel Aviv, Israel

**Keywords:** human embryonic stem cells, neural differentiation, human neural precursor cells, Wnt signal, GSK-3β, tankyrase

## Abstract

The canonical Wnt/β-catenin pathway is a master-regulator of cell fate during embryonic and adult neurogenesis and is therefore a major pharmacological target in basic and clinical research. Chemical manipulation of Wnt signaling during *in vitro* neuronal differentiation of stem cells can alter both the quantity and the quality of the derived neurons. Accordingly, the use of Wnt activators and blockers has become an integral part of differentiation protocols applied to stem cells in recent years. Here, we investigated the effects of the glycogen synthase kinase-3β inhibitor CHIR99021, which upregulates β-catenin agonizing Wnt; and the tankyrase-1/2 inhibitor XAV939, which downregulates β-catenin antagonizing Wnt. Both drugs and their potential neurogenic and anti-neurogenic effects were studied using stable lines human neural precursor cells (hNPCs), derived from embryonic stem cells, which can be induced to generate mature neurons by chemically-defined conditions. We found that Wnt-agonism by CHIR99021 promotes induction of neural differentiation, while also reducing cell proliferation and survival. This effect was not synergistic with those of pro-neural growth factors during long-term neuronal differentiation. Conversely, antagonism of Wnt by XAV939 consistently prevented neuronal progression of hNPCs. We show here how these two drugs can be used to manipulate cell fate and how self-renewing hNPCs can be used as reliable human *in vitro* drug-screening platforms.

## Introduction

The Wnt/β-catenin signaling pathway has been shown to play pivotal roles in embryonic neural development (Le Dreau and Marti, 2012) and adult neurogenesis (Lie et al., 2005). The “canonical” Wnt signaling pathway is activated when the Wnt ligand binds to its membrane receptor generating an intracellular cascade of events, eventually resulting in the escape of β-catenin from its “destruction complex.” Free β-catenin can then translocate from the cytosol to the nucleus, where it acts as a major transcription factor regulating the expression of several important target genes (Cadigan and Peifer, 2009; MacDonald et al., 2009; Zimmerman et al., 2012). Because of its critical role in neurogenesis from early embryonic development to ongoing hippocampal plasticity throughout life, dysregulation of Wnt signaling has been shown to underlie some of the symptoms observed in neurodevelopmental disorders (Luo et al., 2010; Mohn et al., 2014; Zeidan-Chulia et al., 2014; Hormozdiari et al., 2015; Telias et al., 2015b), as well as in psychiatric and neurodegenerative disorders (Oliva et al., 2013; Seib et al., 2013; Hussaini et al., 2014).

β-catenin’ destruction complex is composed of several different proteins, including glycogen synthase kinase-3β (GSK-3β) (Hur and Zhou, 2010; Seira and Del Rio, 2014) and axin (MacDonald et al., 2009; Gammons and Bienz, 2018). Axin is a substrate of tankyrase-1/2 (TANK), a poly-ADP-ribosyltransferase that can target and tag axin for ubiquitination through ribosylation (Huang et al., 2009; Bao et al., 2012). GSK-3β and TANK play multiple Wnt-independent roles in cell biology, including in proliferation, differentiation, and metabolism (Ali et al., 2001; Cohen and Frame, 2001; Seimiya, 2006; Hsiao and Smith, 2008). They both regulate β-catenin activity, but in opposite directions: while GSK-3β activity increases β-catenin phosphorylation and degradation, reducing Wnt signaling; TANK enzymatic activity on axin results in increased levels of free β-catenin, increasing Wnt signaling.

Much effort has been invested in recent years in the development of suitable small-molecule inhibitor drugs targeting GSK-3β and TANK (Riffell et al., 2012; King et al., 2014). In this study, we have assessed the activity of two specific drugs: the amino-pyrimidine CHIR99021 (“CHIR”), a highly specific inhibitor of GSK-3β, with potential applications to cognitive impairment in neurodevelopmental disorders (Franklin et al., 2014) and psychiatric illness (Garza et al., 2018); and the pyrimidine XAV939 (“XAV”), a specific inhibitor of TANK, successfully tested as a promising anti-neoplastic drug (Huang et al., 2009; Bao et al., 2012; Busch et al., 2013). Most relevant to the present report, is the use of CHIR and XAV as critical components of chemically defined protocols aiming to guide differentiation of human and murine stem cells into myriad cellular fates. CHIR has been used to promote neural and neuronal differentiation in embryonic stem cells and induced pluripotent stem cells (De Kumar et al., 2017; Shafa et al., 2018; Gomez et al., 2019; Qiu et al., 2019; Shin et al., 2019; Bejoy et al., 2020) and in several different types of neural precursors (Yang Y. et al., 2020; Ren et al., 2021; Wang et al., 2021), as well as to induce *trans-*differentiation into neural lineages from non-neural cell types, such as skin (Bataille et al., 2020; Yang J. et al., 2020) and mesenchymal cells (Govarthanan et al., 2020). XAV has been used to promote differentiation of pluripotent stem cells into lung cells (Malleske et al., 2018; Kanagaki et al., 2020), to increase osteogenic differentiation of mesenchymal stem cells (Almasoud et al., 2020; Han et al., 2020), and to promote generation of cardiomyocytes (Hamad et al., 2019; Leigh et al., 2020).

In this brief report, we show the effects of chemical manipulation of Wnt using CHIR and XAV on human neural precursor cells (hNPCs), differentiated from human embryonic stem cells (hESCs) (Telias et al., 2015b). These cells can be kept as self-propagating hNPCs for >10 passages, and can be induced to undergo neuronal and glial differentiation by changing medium composition and growth conditions (Telias et al., 2013, 2014, 2015a). The pro- and anti-neural effects of CHIR and XAV were assessed at the molecular level by measuring gene and protein expression, and at the cellular level by implementing a morphological bioassay indicative of neural progression. We also examined the effect of CHIR and XAV on hNPCs survival rate and proliferation. As expected, during long-term neuronal maturation, CHIR had an overall neurogenic effect, while XAV did not, in line with what is known for the role of Wnt during neuronal differentiation. Our results show that CHIR is neurogenic but also probably toxic to hNPCs, while XAV had the opposite effect, reducing neuronal yield and inducing a more primitive developmental phenotype reminiscent of undifferentiated hESCs. This study exemplifies the value of using *in vitro* drug screening platforms based on stem cells and their derivatives, emphasizing their dynamism and malleability.

## Materials and Methods

### Human Embryonic Stem Cells

Four non-affected hESC lines were obtained from Harvard University Stem Cell Core, including HUES-6 (XX), HUES-16, HUES-13, and HUES-64 (XY) (Cowan et al., 2004; Osafune et al., 2008; Bock et al., 2011; Telias et al., 2015b). Undifferentiated hESC colonies were cultured on Matrigel (BD)-coated polystyrene wells, in the presence of inactivated mouse embryonic fibroblasts (MEFs), in liquid medium composed of DMEM:F12, supplemented with 20% knock-out serum replacement, 1% Glutamax, 1% insulin transferrin selenium (all purchased from LifeTech.), 1% non-essential amino-acids (BioInd.), and 50 ng/ml Primocin (InvivoGen) (Telias et al., 2013, 2014, 2015b). Cell medium was refreshed every 48 h and supplemented with 8 ng/ml basic fibroblast growth factor (bFGF, R&D) to maintain pluripotency and prevent spontaneous differentiation *in vitro*.

### Derivation of Human Neural Precursor Cells and Neuronal Differentiation

*In vitro* neural differentiation (IVND) of hESCs was carried out as previously described (Telias et al., 2013, 2015b). In brief, hESCs were grown in Neural Induction Medium (NIM) consisting of DMEM:F12, 0.5% B27 supplement, 1% N2 supplement, 1% Glutamax, 1% non-essential amino acids and 0.1 mg/ml Primocin. IVND included three steps: (a) formation of neuro-ectoderm in the presence of 250 ng/ml noggin (PeproTech) and 20 ng/ml bFGF; (b) development of neural rosettes in the presence of 200 ng/ml sonic hedgehog (Shh, PeproTech) and (c) generation of neurospheres aggregates by manual trituration, re-suspended in NIM containing 20 ng/ml bFGF.

To induce the formation of self-renewable hNPCs, floating neurospheres were re-seeded on Matrigel-coated polystyrene in NIM with 20 ng/ml bFGF. Self-renewing hNPCs were grown for a minimum of 4 and a maximum of 12 passages before initiating any experiment. Cultures were passaged once a week at a dilution ratio of 1:6. For passaging, cells were dissociated using TryplE (LifeTech) at 37°C for 2–3 min. Cells were gently pipetted, collected and centrifuged in a conical 15-ml tube (5′, 1,200 RPM) before re-seeding.

Neuronal differentiation of hNPCs was induced by cells dissociation using TryplE and re-plating onto Poly-D-Lysine/Laminin (Sigma)-coated glass coverslips. NIM was replaced with Neuronal Differentiation Medium (NDM) supplemented with brain-derived neurotrophic factor (BDNF), glia-derived neurotrophic factor (GDNF) and neurotrophin-3 (NT-3, all 10 ng/ml, PeproTech). NDM composition was similar to NIM, but using Neurobasal (LifeTech) instead of DMEM:F12.

### Pharmacological Manipulation of Wnt Signaling

The GSK-3β inhibitor CHIR99021 {6-[[2-[[4-(2,4-Dichlorophenyl)-5-(5-methyl-1H-imidazol-2-yl)-2-pyrimidinyl] amino]ethyl]amino]-3-pyridinecarbonitrile; a.k.a. “CHIR”} was purchased from Tocris (Cat #4423). The TANK inhibitor XAV939 {2-[4-(trifluoromethyl)phenyl]-1,5,7,8-tetrahydrothiopyrano[4,3-d]pyrimidin-4-one; a.k.a. “XAV”} was obtained from Selleckchem (Cat #A1877). Both compounds were dissolved in dimethyl sulfoxide (DMSO) at 20 mM stock solutions and stored at −80°C, in the dark. Fresh CHIR or XAV was added with every medium change, every 48–72 h. Working concentrations of both pharmacological agents was 3 μM in all experiments (De Kumar et al., 2017; Major et al., 2017; Shafa et al., 2018; Srikanth et al., 2018), resulting in a DMSO content of 0.015%. Accordingly, all control experiments, without CHIR or XAV, included 0.015% DMSO.

### Gene Expression Analysis

Relative transcription levels were analyzed by quantitative RT-PCR, as previously described (Telias et al., 2013, 2015b). RNA was extracted (RNeasy, Qiagen), reversed transcribed using Super Script-III kit (Invitrogen), and analyzed using SYBRgreen (ABgene) in Rotor Gene 6000 Series (Corbett). The house keeping gene GAPDH was used as a control for ΔΔCt analysis. All qRT-PCR assays included non-template control and non-human cells control (MEFs). Primer sequences (5′–3′) were as follows: GAPDH, R-atacgaccaaatccgttgactc, F-agccacatcgctcagacacc; MAP2, R-cattggcgcttcggacaag, F-ctcag caccgctaacagagg; GFAP, R-aggtccatgtggagcttgac, F-gccattgcc tcatactgcgt; TUJ1, R-tttttgctcgcctcaaggtatgt, F-gggcgcattccaacctt; TAU, R-tgccatgttgagcaggacta, and F-tcacttttacagcaacagtcagtg. All qRT-PCR experiments included a non-human negative control (RNA extracted from MEFs) and a no-template control (no DNA template in the reaction).

### Western Blot Analysis

Western blot analysis was carried out as previously described (Telias et al., 2015b). Protein was extracted using reporter lysis buffer (Promega), and 25–30 μg of protein were loaded on a 7.5% separating gel using Mini *Trans-*Blot Cell (Bio-Rad). Nitrocellulose membranes were stained with primary antibodies against human β-catenin (Santa Cruz, #sc7199, 1:250 dilution) and human β-Actin (Abcam, #ab8224, 1:500 dilution), and detected with HRP-conjugated secondary antibodies (Jackson ImmunoResearch, 1:10,000 dilution). Protein bends were detected using EZ-ECL (BioInd.). Gel images were analyzed using ImageJ (NIH). Identical regions of interest were drawn around β-catenin and β-Actin gel bands across samples and cell lines, and mean gray value was measured in all of them.

### Live Cell Imaging and Morphological Assay

Live cells were imaged in NIM or NDM (refracting medium) while growing on glass coverslips coated in Poly-D-Lysine/Laminin (Sigma) placed at the bottom of polystyrene wells. Images were taken using 10× and 40× objectives on an inverted light microscope (Olympus XI-50), in bright field. During imaging, the plate cover was not removed, to prevent contamination. Systematic imaging of live hNPCs and neurons during neural induction and neuronal differentiation were obtained in all four lines employed in the study, at regular time intervals: every 2 days during the 7 days of neural induction, and every 5 days during the 30 days of neuronal differentiation. Morphological analysis was conducted *post hoc* using Cell^A (Olympus) and ImageJ software. Three representative images for each condition and developmental stage were sampled by manually drawing cellular contours on bright field images. The total surface area and perimeter included in each contour was automatically provided by the software after setting the image scale in μm. A minimum of 50 cells were chosen for morphological analysis in every image.

### Immunofluorescence and Proliferation Assay

Immunostaining and immunofluorescent imaging was performed as previously described (Telias et al., 2013, 2015b). Cells were seeded on glass coverslips coated with Matrigel for hNPCs or with Poly-D-Lysine/Laminin for neuronal differentiation. Cells were fixated using Cytofix (BD). *Proliferation rate in hNPCs*: cells were labeled using a primary antibody against human Ki67 (R&D, #MAB7617, 1:500 dilution) and counterstained with the nuclear dye DAPI (Sigma) before mounting. *MAP2-GFAP assay in neurons*: cells were double-labeled using primary antibodies against human MAP2 (Santa Cruz, #sc-20172, 1:250 dilution) and human GFAP (Millipore, #MAB360, 1:250 dilution). All immunofluorescence was performed over-night at 4°C and detected using Cy2/Cy3-conjugated secondary antibodies (LifeTech., 1:1,000 dilution). Coverslips were mounted on glass slides using Fluoromount G (Southern Biotech). Cells were imaged using an inverted fluorescent Olympus XI-50 microscope. All conditions were similar for all lines in all experiments, every experiment was repeated three times in each of the four cell lines. In each coverslip, images were collected in three randomly selected fields, each field measuring 200 × 200 μm. Images were used *post hoc* to count the number of Ki67-positive cells and the total number of cells (DAPI), or to measure mean gray value of Cy2/Cy3 in MAP2-GFAP assay. Image analysis was conducted using ImageJ software (NIH).

### Survival Assay

Human neural precursor cells were seeded at low density (30,000 cells p/well in a 24-well plate) and incubated for 7 days with either CHIR99021 or XAV939 (3 μM). Medium was refreshed every 48 h. At the end of 7 days, living cultures were detached from their wells and dissociated to single cells using TryplE (LifeTech). Dissociated cells were centrifuged at 1,200 RPM for 5 min in 15-ml conical tubes. Cells were mounted onto a standard hemocytometer and manually counted.

### Statistical Analysis

Statistical analysis (Student’s *t*-test and ANOVA) was performed using SPSS, SigmaPlot and online GraphPad QuickCalcs.^1^

## Results

### Experimental Setup

**FIGURE 1:**
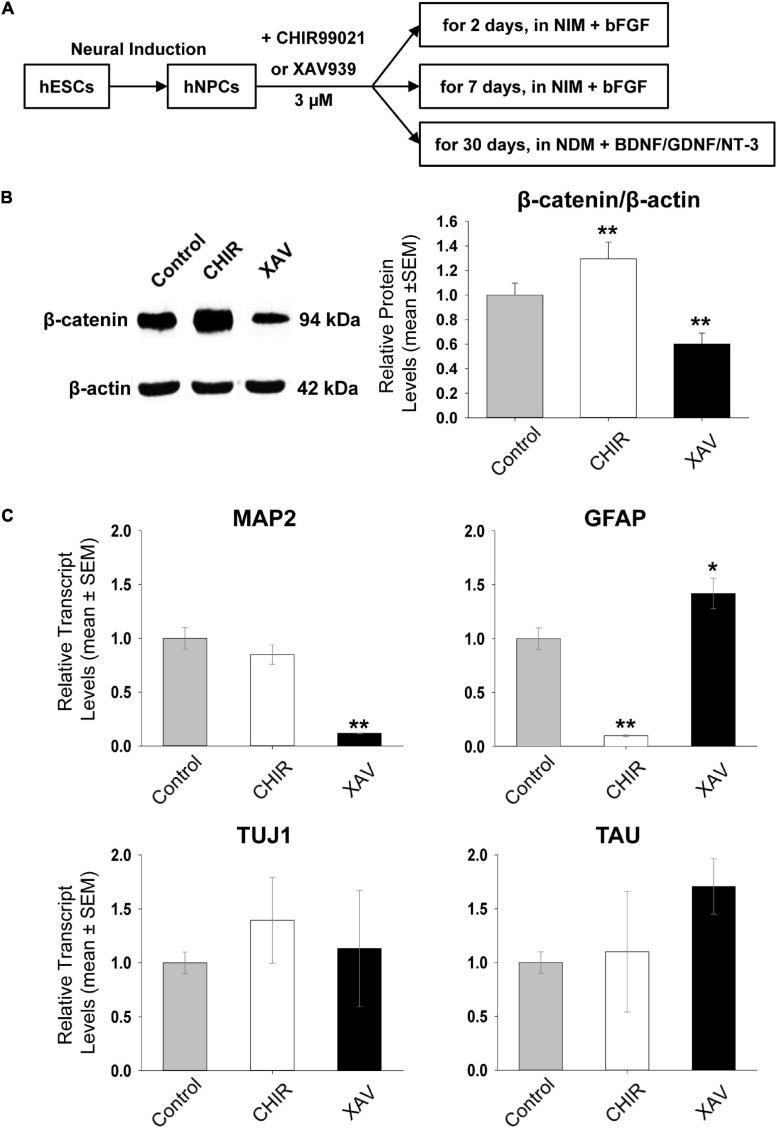
Short-term effect of Wnt modulators on hNPCs. **(A)** Schematic outline of the experimental set-up. Human embryonic stem cells (hESCs) are differentiated into neural precursor cells (hNPCs), used to test the effects of 3 μM CHIR99021 (CHIR) or XAV939 (XAV). hNPCs were either maintained in NIM containing basic fibroblast growth factor (bFGF), or in Neural Differentiation Medium (NDM) containing brain-derived neurotrophic factor (BDNF), glia-derived neurotrophic factor (GDNF) and neurotrophin-3 (NT-3). **(B)** Western blot analysis of β-catenin protein levels after treating hNPCs in NIM containing bFGF with 3 μM CHIR or XAV for 2 days. β-actin was used as loading and normalizing control. *Left* representative images for Western blots, obtained in HUES13-hNPC line. Gel images were used to establish mean gray value (MGV) in regions of interest of equal surface size (densitometry). *Right* Quantification of β-catenin/β-actin MGV ratio, normalized to control (control: gray bars, CHIR: white bars, XAV: black bars). Values are mean ± SEM. Experiments repeated three times in 4 hNPC lines, **p < 0.01, ANOVA. **(C)** Effect of treatment with 3 μM CHIR or XAV for 2 days on the transcriptional status of neural genes. qRT-PCR measurement of MAP2, GFAP, TUJ1, and TAU relative transcript levels (“ΔΔCt”), using the housekeeping gene GAPDH as an internal control (control: gray bars, CHIR: white bars, XAV: black bars). Experiments repeated three times in 4 hNPC lines. Values are mean ± SEM, *p < 0.05; **p < 0.01, ANOVA.

In this brief report we tested the effects of CHIR, a specific GSK-3β inhibitor and XAV, a specific TANK inhibitor, on cultured hNPCs used as a human *in vitro* drug screening platform. All four hNPC lines used in this study were previously derived from four different hESC lines (Telias et al., 2015b). The effects of CHIR and XAV on hNPCs were analyzed following short-term (2 days), mid-term (7 days), or long-term treatment (30 days; Figure 1A) with either drug as compared to non-treated controls, using gene and protein expression analysis and cell-based assays as readouts. Short- and mid-term experiments were conducted utilizing self-renewing hNPCs kept in chemically defined NIM medium (see “Materials and Methods” section), formulated to promote self-propagation and to halt developmental progression into mature neurons, by supplementing it with basic fibroblast growth factor (bFGF). Long-term treatment of 30 days was concomitant with active neuronal differentiation of hNPCs, achieved by removing bFGF and switching medium to NDM supplemented with three neurotrophic growth factors. In all cases, either drug was used at a final concentration of 3 μM, directly dissolved in cell media and refreshed every 48 h.

### Immediate Effects of Wnt Modulators on Self-Renewing hNPCs

To investigate the potential neurogenic or anti-neurogenic effect of CHIR and XAV on self-renewing hNPCs growing on Matrigel in the presence of bFGF, we first confirmed that both drugs are indeed able to affect the protein levels of β-catenin. GSK-3β phosphorylates two different serine residues on the N-terminus of β-catenin, directly tagging it for ubiquitination and degradation. TANK indirectly increases β-catenin levels by de-activating Axin, an important member of the destruction complex behind β-catenin degradation. Approximately 1 million cells per well were incubated with either drug at 3 μM for 48 h. They were then harvested for protein purification and analyzed using Western blot. As expected, incubation of hNPCs with CHIR led to a significant increase in β-catenin protein levels and similar treatment with XAV to a significant decrease (control = 100 ± 9.7%, CHIR = 129.5 ± 13.7%, XAV = 60.25 ± 8.9, *p* < 0.01, *n* = 3/line, 4 hNPC lines, Figure 1B). The expression of β-catenin was normalized to the expression of the housekeeping gene β-Actin. No noticeable changes in culture density or cellular morphology were observed during short-term exposure to CHIR or XAV.

We also measured whether 48 h of hNPCs exposure to CHIR or XAV was sufficient to induce changes in gene expression reflecting a change in their neurodevelopmental status. We have previously shown that MAP2, GFAP, TUJ1, and TAU, are all expressed in self-renewable hNPCs under continuous presence of bFGF (Telias et al., 2013, 2015b). The expression of these markers increases quantitatively as hNPCs progress into more mature neuronal cell types (Telias et al., 2015b), such as neuroblasts, and finally segregate qualitatively in mature neurons (MAP2, TUJ1, and TAU) and glial cells (GFAP).

Human neural precursor cells were harvested following 48 h of incubation with 3 μM CHIR or XAV, and their RNA purified. Using quantitative Real-Time PCR, we measured the expression of MAP2, GFAP, TUJ1, and TAU relative to that of the housekeeping gene GAPDH (Figure 1C). Our results show that treatment of hNPCs with CHIR or XAV was able to affect the overall transcription of MAP2 and GFAP but not that of TUJ1 or TAU. Short-term exposure to CHIR reduced GFAP in hNPCs (11.4 ± 3.0% of control, *p* < 0.01, *n* = 3/line, 4 hNPC lines) but did not significantly alter the expression of MAP2. XAV had opposite effects in GFAP and MAP2 expression, significantly increasing GFAP (142.0 ± 15% of control, *p* < 0.05, *n* = 3/line, 4 hNPC lines) and significantly reducing MAP2 (12.3 ± 9.4% of control, *p* < 0.01, *n* = 3/line, 4 hNPC lines). These results suggest that 48 h of GSK-3β inhibition by CHIR is not sufficient to induce neuralization of hNPCs, while inhibition of TANK by XAV under the same conditions is effective in inducing an anti-neural response, decreasing MAP2 and increasing GFAP. These results further suggest that while MAP2 and GFAP transcriptional activation in hNPCs might be subjected to modulation by Wnt/β-catenin, TUJ1 and TAU seem to be unaffected by it in these cells.

### Sustained Wnt Agonism and Antagonism in Self-Renewing hNPCs

**FIGURE 2:**
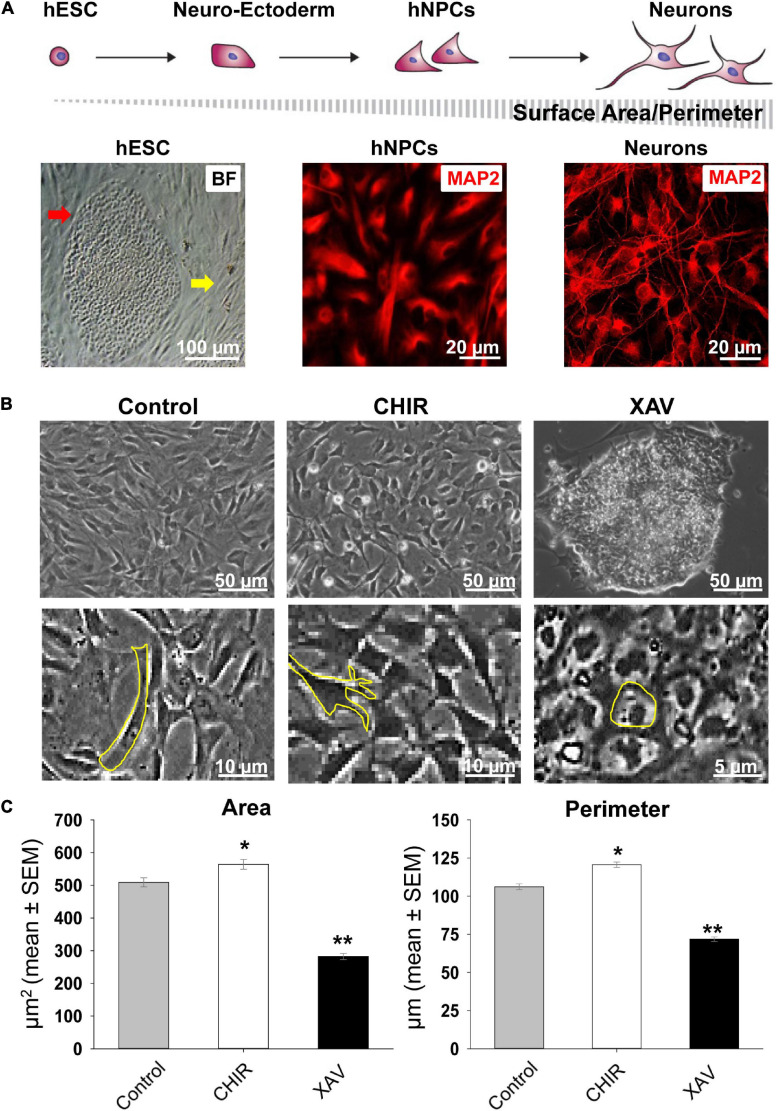
Changes in neural morphology induced by Wnt modulators. **(A)** Top row: schematic presentation of the morphological changes in cells from undifferentiated hESCs to mature neurons during *in vitro* neural differentiation, and how they affect the relative change in surface area and perimeter. *Bottom row*, from left to right: bright field (BF) image of a single fixed undifferentiated HUES13-hESC colony (red arrow) surrounded by inactivated mouse embryonic fibroblasts (yellow arrow); immunofluorescence image of fixed hNPCs stained with MAP2 (red) and similar staining in mature neurons derived from HUES-13. **(B)** Top row: representative images of living cultures of HUES-64 hNPCs in control conditions (NIM + bFGF) and after 7 days of treatment with 3 μM CHIR or XAV. *Bottom row*: magnified images exemplifying contour determination for the calculation of surface area and perimeter in morphological assays. **(C)** Quantification of soma area (μm^2^) and cell perimeter (μm) of hNPCs (living cells, non-fixed) in control conditions (gray bars), and following treatment with 3 μM CHIR (white bars) or XAV (black bars) for 7 days. Experiments were repeated three times in all 4 hNPC lines, 3 random fields were imaged in each experiment, >50 cells were analyzed in each field. Values are mean ± SEM, *p < 0.05; **p < 0.01, ANOVA.

Next, we tested the response of hNPCs to 7 days-long treatments with CHIR or XAV. As hNPCs are kept in a state of self-renewal by bFGF, these experiments uncover whether CHIR or XAV can “push” these cells into a different developmental state despite the presence of bFGF. We tracked their potential neural differentiation by implementing a morphological assay in live cultures (Figure 2A), based on the premise that, as neural differentiation progresses, cells become bigger and produce more numerous and longer projections, showing an increase in total surface area and perimeter. Using specialized software, individual cells in image samples were used to delineate their contour, creating regions of interest from which perimeter (in μm) and area (in μm^2^) were then calculated (Figure 2B). Our results show that exposing hNPCs to CHIR for 7 days conferred them a more neuronal phenotype, increasing the number of neurite projections and their length (Figure 2C). In contrast, XAV elicited the opposite effect, conferring cells an almost undifferentiated, hESC-like morphology, with small round somata and with no membrane projections. Specifically, CHIR increased the mean cell area from 509.1 ± 13.9 to 564.1 ± 15.1 μm^2^ (*p* < 0.05, >50 cells/experiment, *n* = 3/line, 4 hNPC lines), while XAV reduced it to 282.2 ± 9.4 μm^2^ (*p* < 0.01, *n* same as above). Their effect on mean cell perimeter was similar: CHIR increased cellular perimeter from 106.2 ± 2.0 to 120.6 ± 1.7 μm (*p* < 0.05) and XAV reduced it to 71.8 ± 1.5 μm (*p* < 0.01).

### Effect of Wnt Modulators on hNPCs Proliferation and Survival

**FIGURE 3:**
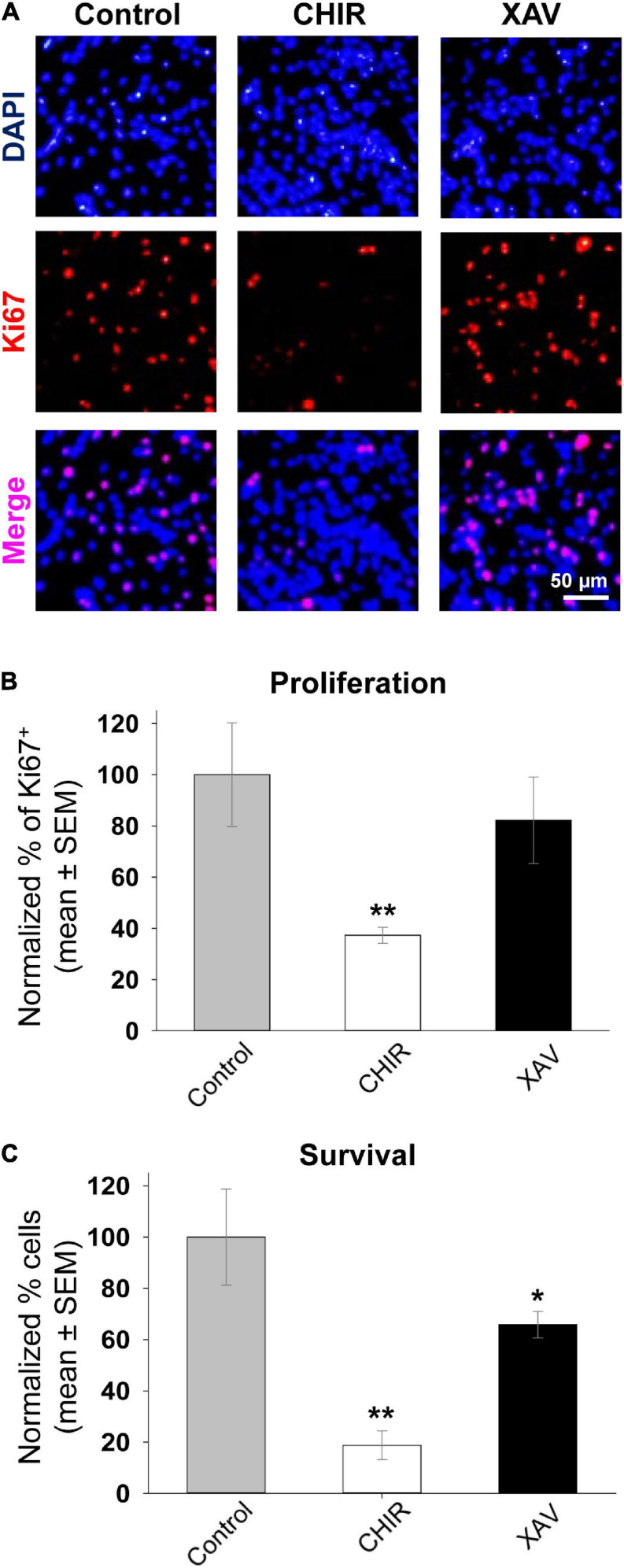
Effect of Wnt modulators on proliferation and survival of hNPCs. **(A)** Representative images of fixed HUES-6 hNPCs in control conditions (NIM + bFGF) and after 7 days of treatment with 3 μM CHIR or XAV, stained with the nuclear dye DAPI (*top row*, blue) and immunostained with the proliferation marker Ki67 (*middle row*, red). Bottom row shows the merged images of DAPI and Ki67 double staining (pink). **(B)** Proliferation rate quantified as the percentage of Ki67-positive cells from the total number of cells (DAPI-positive), in control conditions (gray bar) and following hNPCs exposure to 3 μM of CHIR (white) or XAV (black) for a total of 7 days. Experiments were repeated three times in all 4 hNPC lines, 3 random fields were imaged and analyzed in each experiment. Quantification was conducted *post hoc* using ImageJ ROI tool. Values are shown as mean ± SEM, normalized to control. *p < 0.05; **p < 0.01, ANOVA. **(C)** Survival assay conducted on hNPCs after 7 days of control conditions (gray bar) or treatment with 3 μM CHIR (white bar) or XAV (black bar). At time = 0 days, ∼30,000 cells were seeded on 24-well plates. At time = 7 days, the number of cells in culture was manually counted using a hemocytometer. Experiments were repeated three times in all 4 hNPC lines. Values are shown as mean ± SEM, normalized to control. *p < 0.05; **p < 0.01, ANOVA.

Most studies using CHIR or XAV to guide differentiation of stem cells and other cell types used a final concentration of 2.5–10 μM (De Kumar et al., 2017; Lancaster et al., 2017; Major et al., 2017; Shafa et al., 2018; Sharma et al., 2018; Srikanth et al., 2018; Yoon et al., 2019; Bataille et al., 2020). Yet, the same studies have not addressed whether these drugs have any cytotoxic effects on human or murine cell cultures. We measured proliferation and survival of hNPCs when grown in the presence of bFGF and treated with 3 μM CHIR or XAV for 7 days, refreshing cell medium every 48 h. To measure proliferation, we fixed and stained sample cultures belonging to all four hNPC lines after 7 days of treatment and carried out immuno-fluorescence assays to detect and quantify the presence of the proliferation marker Ki67 (Figure 3A). Quantification of Ki67 expression, normalized to the total number of cells revealed with the nuclear dye DAPI, showed that treatment with CHIR resulted in a significantly reduced proliferation rate, whereas treatment with XAV did not (Figure 3B). The expression of Ki67 in CHIR-treated hNPCs was 37.3 ± 3.1% of that found in control counterparts (*p* < 0.01, >50 cells/experiment, *n* = 3/line, 4 hNPC lines). Following the same experimental setup of a 7 days-long treatment with CHIR or XAV, we carried out a complementary survival assay, in which hNPCs were first seeded at low densities in Matrigel-coated polystyrene wells, then submitted to the treatment, and finally harvested. The total cell count at day 0 before seeding was compared to the total cell count per well at the end of treatment at day 7 (Figure 3C). The results show that CHIR induces a robust reduction in cell survival (*p* < 0.01), and XAV a more modest but still significant reduction too (*p* < 0.05). Taken together, the data shown in Figures 2, 3 suggest that CHIR may act as a pro-neural agent in hNPCs, increasing neural differentiation and reducing proliferation, but at the same time it might be toxic to cells. In contrast, XAV acts as an anti-neurogenic agent, without significantly altering the cell proliferation rate and is less toxic at the same concentration *in vitro*. Given these results, we suggest that differentiation protocols including CHIR or XAV should include tests addressing possible cytotoxicity by these compounds.

### Effect of Wnt Modulators on Neuronal Differentiation of hNPCs

**FIGURE 4:**
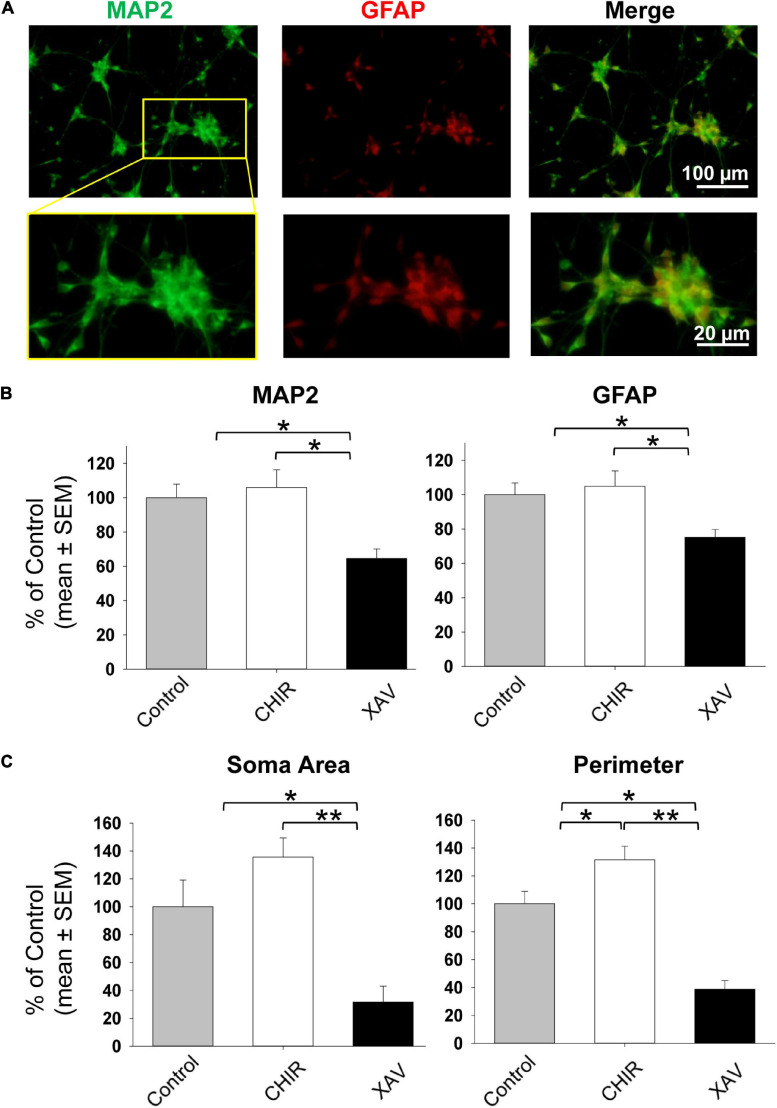
Neuronal differentiation of hNPCs treated with Wnt modulators. **(A)** Representative images of fixed hNPCs-derived neurons after 30 days of neuronal differentiation, immunostained for MAP2 (green) and GFAP (red). Top row: full field of view. Bottom row: enhanced detail of a seeded neurosphere with MAP2 and GFAP positive cells protruding outward. Neuronal differentiation was induced by detaching hNPCs and re-seeding neurosphere onto poly-D-lysine/laminin coated glass coverslips. Cells were grown in neural differentiation medium (NDM) including BDNF, GDNF, and NT-3 (see “Materials and Methods” section). **(B)** Quantification of MAP2 and GFAP immunostaining as shown in panel **(A)**, following 30 days on neuronal differentiation in control conditions (gray bars), or under treatment with 3 μM CHIR (white bars) or XAV (black bars). Experiments were repeated three times in all 4 hNPC lines. Values are shown as mean ± SEM, relative to control. *p < 0.05, ANOVA. **(C)** Morphological assessment of neuronal differentiation (see also Figures 2B,C). The soma area (*left*) and the perimeter (*right*) of living cells was quantified in control conditions (gray bars), or after treatment with 3 μM CHIR (white bars) or XAV (black bars). Experiments were repeated three times in all 4 hNPC lines, 3 random fields were imaged in each experiment, >50 cells were analyzed in each field. Values are shown as mean ± SEM, relative to control. *p < 0.05; **p < 0.01, ANOVA.

The possible pro- and anti-neural effects of 2–7 days of treatment with CHIR and XAV on self-renewing hNPCs might be interpreted as the net effect when weighing their tendency to remain in their developmental state on one hand, against the ability of these drugs to effectively induce a developmental change on the other. Therefore, we induced neuronal maturation of hNPCs for 30 days, by removing bFGF and adding neuronal growth factors (Figure 1A), previously shown to guide hESC and hNPC differentiation into mature glutamatergic neurons (Telias et al., 2013, 2015a). Neuronal differentiation medium, pro-neural growth factors, and Wnt manipulators were refreshed every 3 days. At the end of the 30 days process, sample cultures were fixed and immune-stained with anti-MAP2 and anti-GFAP antibodies (Figure 4A). We then quantified the relative amount of MAP2 and GFAP fluorescence in all conditions (Telias et al., 2013, 2015b), for all four cell lines employed in the study. Our results show that 30 days of neuronal differentiation in the presence of CHIR did not significantly increase the levels of MAP2 or GFAP above control counterparts (Figure 4B), but XAV treatment significantly reduced the expression of both markers (MAP2 64.5 ± 5.6% of control, GFAP 75.2 ± 4.6, *p* < 0.05, >50 cells/experiment, *n* = 3/line, 4 hNPC lines). Employing the same live-cell imaging-based morphological assessment of neural differentiation as shown in Figure 2, we found that treatment with CHIR did not significantly affect the area of somata (135.6 ± 13.7% of control, *p* > 0.05, >50 cells/experiment, *n* = 3/line, 4 hNPC lines, Figure 4C), but significantly increased their perimeter, reflecting the increase in projection number and length (131.5 ± 9.8% of control, *p* < 0.05). Conversely, XAV robustly reduced both the soma area and the total perimeter of cells (area: 31.7 ± 11.4% of control; perimeter: 38.8 ± 6.2% of control, *p* < 0.01). These results confirm the neurogenic effect of CHIR and the anti-neurogenic effect of XAV during the time-course of neuronal differentiation, as expected.

## Discussion and Conclusion

In this report we investigated the effects of Wnt modulation on the neural status and neuronal differentiation of hNPCs derived from hESCs. The Wnt activator CHIR and the Wnt inhibitor XAV were tested on self-renewable hNPCs, without inducing neuronal differentiation, in two modalities: after only 48 h of single treatment, and after 7 days of continuous treatment. We observed that the Wnt activator CHIR was able to induce a more neuronal-like morphology in these cells, against the presence of bFGF which promotes self-renewal and delays neural progression. Once neuronal maturation was induced by removing bFGF and changing cell growing conditions to those promoting neuronal differentiation, CHIR modestly enhanced neuronal fate determination, in synergism with the effects of BDNF, GDNF, and NT-3. We conclude that, as expected (Bengoa-Vergniory and Kypta, 2015), Wnt activation working via enhancement of β-catenin activity, seems to promote neuralization of hNPCs.

Counterpart experiments using the Wnt inhibitor XAV showed, as expected, the opposite effect. In the presence of bFGF, stable hNPCs treated with XAV adopted a morphology more reminiscent of undifferentiated hESCs or epithelial cells, lacking membrane projections or spindle-like somata, characteristic of hNPCs, neuroblasts and early neurons. Whereas XAV induced de-differentiation of hNPCs back to hESCs, or *trans-*differentiation into endoderm- o mesoderm-like lineages, is out of the scope of this study. Induction of neuronal differentiation of hNPCs for 30 days in the presence of XAV resulted in significantly reduced levels of MAP2 and GFAP expression, and in cells lacking any microstructure identifiable as neuronal, including neurites or axons. In line with previous knowledge (Bengoa-Vergniory and Kypta, 2015), we conclude that inhibition of Wnt prevents neuronal maturation of hNPCs despite the presence of neuronal growth factors that favor *in vitro* generation of neurons.

Taking advantage of our *in vitro* drug screening platform of self-renewable hNPCs, we also asked whether CHIR or XAV might be cytotoxic, by measuring cell proliferation and survival. Both drugs were used in this study at the same concentration or lower as in similar studies employing human or murine ESCs, hiPSCs and other types of stem cells (De Kumar et al., 2017; Malleske et al., 2018; Shafa et al., 2018; Gomez et al., 2019; Hamad et al., 2019; Qiu et al., 2019; Shin et al., 2019; Almasoud et al., 2020; Bataille et al., 2020; Bejoy et al., 2020; Govarthanan et al., 2020; Han et al., 2020; Kanagaki et al., 2020; Leigh et al., 2020; Yang J. et al., 2020; Yang Y. et al., 2020; Ren et al., 2021; Wang et al., 2021). When stem cells differentiate into more mature cell types, their proliferation potential is gradually reduced, until cells reach a post-mitotic fate. In addition, cells that fail to differentiate usually die and are lost with subsequent medium changes. Therefore, it is no surprise that most chemically defined neural and neuronal differentiation protocols result in cultures with far less cells than the original numbers of precursors present at the beginning of the process. However, in our hands, we observed a reduction of ∼70% in proliferation and ∼80% in survival of hNPCs when treated with CHIR for 7 days, suggesting that CHIR might be toxic to these cells. Despite the growing number of studies in recent years utilizing CHIR to induce neuronal differentiation, a systematic study of its potential cytotoxicity is still lacking, and only two studies addressed this question using different cellular paradigms and readouts. Naujok et al. (2014), using undifferentiated mouse ESCs, showed that a treatment of 3 days with 2.5 μM CHIR reduced cell viability by ∼25%, and increasing CHIR concentration to 5 μM or more, reduced cell viability by >50%. Tu et al. (2017), employing hiPSCs, observed a pro-apoptotic synergistic effect of 8 μM CHIR and different thiol-containing antioxidants, that are otherwise non-toxic when GSK-3β is not being inhibited. A comparison between these studies and ours seems to suggest that CHIR toxicity increases with more advanced differentiation of stem cells, but the scarcity of similar studies and the different conditions under which each study conducted its measurements preclude the drawing of meaningful conclusions. In any case, we suggest that all studies using CHIR as an inducer of neural or neuronal differentiation should also include direct or indirect measurements of proliferation, survival, apoptosis, and cell viability.

Finally, we should mention that studies using human stem cells, both hESCs and hiPSCs, seem to confirm CHIR’s role in promoting neuronal differentiation (Li et al., 2011; Chambers et al., 2012; Denham et al., 2012; Esfandiari et al., 2012; Titmarsh et al., 2012), similar to one study conducted in iPSCs derived from monkeys (Xi et al., 2012). However, in rodent-based models, this was inconsistent. Two studies reported that in rat-derived ESCs and in MEFs, CHIR promoted neural differentiation similar to the human models (Peng et al., 2013; Cheng et al., 2014), but two other studies showed that in mouse ESCs CHIR inhibits neural differentiation, enhancing pluripotency-maintenance mechanisms (Ye et al., 2012; Wu et al., 2013). This discrepancy between human and murine *in vitro* platforms, highlights the importance of the use of human-based models in neurodevelopmental research and in *in vitro* drug screening.

## Data Availability Statement

The original contributions presented in the study are included in the article/supplementary material, further inquiries can be directed to the corresponding author.

## Author Contributions

MT designed and executed the experiments, acquired and analyzed the data, and wrote the manuscript. DB-Y conceptualized the study and supervised it, secured funding, and wrote the manuscript. Both authors contributed to the article and approved the submitted version.

## Conflict of Interest

The authors declare that the research was conducted in the absence of any commercial or financial relationships that could be construed as a potential conflict of interest.

## Publisher’s Note

All claims expressed in this article are solely those of the authors and do not necessarily represent those of their affiliated organizations, or those of the publisher, the editors and the reviewers. Any product that may be evaluated in this article, or claim that may be made by its manufacturer, is not guaranteed or endorsed by the publisher.

## Footnotes

1
http://www.graphpad.com/quickcalcs
